# Supplementary material for: Government influence on e-government adoption by citizens in Colombia: Empirical evidence in a Latin American context
Source: PLoS One. 2022 Feb 25;17(2):e0264495. doi: 10.1371/journal.pone.0264495 (PMC8880567; doi:10.1371/journal.pone.0264495)
Supplement: S1 Appendix — Note. Promoting e-government for the current term (PE-CT); Promoting e-government for past due debts (PE-PDD); Promoting physical office for past due debts (PPO-PDD); Conform to the environment (C), mobilization (M), and laws and regulations (L). (DOCX) [file pone.0264495.s001.docx]

| **Week** | **Date** | **Description** | **Factor** |
| --- | --- | --- | --- |
| 108 | 2/24/2018 | Web portal update for usability and reliability. Mobile APP update for usability, security, and information quality. | C |
| 108 | 2/24/2018 | Start of the campaign promoting services through the mobile APP. | M |
| 109 | 3/9/2018 | Web portal update for usability and reliability. | C |
| 110 | 3/12/2018 | Five days of advertising in social media (Google, Facebook, and Instagram), promoting the payment with a 10% discount. Ad clicks: 29,422. Visits to the web platform: 134,445. | M |
| 110 | 3/16/2018 | Early payment deadline for 10% discount. Mandate 72 of December 27, 2017, from the Departmental Assembly of Antioquia. | L |
| 123 | 6/13/2018 | Web portal update for privacy and usability. | C |
| 127 | 7/13/2018 | Deadline for the declaration and payment. Mandate 72 of 27 December 2017, from the Departmental Assembly of Antioquia. | L |
| 138 | 9/28/2018 | Web portal update including training video for users. | C |
| 138 | 9/28/2018 | Web portal update including measuring user satisfaction. | C |
| 150 | 12/18/2018 | Mobile APP update for usability. | C |
| 204 | 2/01/2019 | Web portal update for interoperability with SAP for information quality. | C |
| 205 | 2/05/2019 | Web portal update for usability. | C |
| 207 | 2/22/2019 | Social media (Google, Facebook, and Instagram) advertising for 31 days, promoting the 10% payment discount. Ad clicks: 25,130. Visits to the web platform: 330,837. | M |
| 208 | 2/26/2019 | Web portal update for usability and reliability. | C |
| 208 | 2/27/2919 | Mobile APP update for usability and reliability. | C |
| 211 | 3/22/2019 | Early payment deadline for 10% discount. Mandate 43 of 17 December 2018, from the Departmental Assembly of Antioquia | L |
| 212 | 3/27/2019 | Mandate 9 of 27 March 2019, from the Departmental Assembly of Antioquia, establishing a 70% discount until 31 October 2019, on interest and penalties for past due debts in judicial collection (less than 20 cases had applied). | L |
| 213 | 4/1/2019 | Web portal update for reliability and feedback to the user on transactions. | C |
| 218 | 5/10/2019 | SMS campaign to 48,602 vehicles PE-PDD. | M |
| 219 | 5/13/2019 | Social media (Google, Facebook, and Instagram) advertising for 68 days, promoting the payment. Ad clicks: 88,181. Visits to the web platform: 367,064. | M |
| 219 | 5/14/2019 | SMS campaign to 45,465 vehicles PE-CT. | M |
| 219 | 5/16/2019 | SMS campaign to 118,010 vehicles PE-CT. | M |
| 220 | 5/20/2019 | SMS campaign to 11,606 vehicles PE-PDD. | M |
| 220 | 5/22/2019 | SMS campaign to 8,384 vehicles PE-PDD. | M |
| 220 | 5/23/2019 | SMS campaign to 64,703 vehicles PE-CT. | M |
| 220 | 5/24/2019 | SMS campaign to 5,306 vehicles PPO-PDD. | M |
| 221 | 5/27/2019 | SMS campaign to 97,933 vehicles PPO-PDD. | M |
| 221 | 5/28/2019 | SMS campaign to 5,665 vehicles PE-PDD. | M |
| 221 | 5/29/2019 | SMS campaign to 2,627 vehicles related to past due debts, promoting payment. | M |
| 221 | 5/30/2019 | SMS campaign to 5,689 vehicles PE-PDD. | M |
| 221 | 5/31/2019 | SMS campaign to 55.490 vehicles PE-CT. | M |
| 222 | 6/4/2019 | SMS campaign to 2,576 vehicles PPO-PDD. | M |
| 222 | 6/5/2019 | SMS campaign to 129.910 vehicles PE-CT. | M |
| 222 | 6/7/2019 | SMS campaign to 24,214 vehicles PE-PDD. | M |
| 223 | 6/10/2019 | SMS campaign to 71,387 vehicles PE-CT. | M |
| 223 | 6/11/2019 | SMS campaign to 12,333 vehicles PE-PDD. | M |
| 223 | 6/12/2019 | SMS campaign to 10,000 vehicles PE-CT. | M |
| 223 | 6/14/2019 | SMS campaign to 10,001 vehicles PE-CT. | M |
| 223 | 6/14/2019 | Web portal update for reliability and facilitating conditions (artificial intelligence-based assistant). | C |
| 224 | 6/18/2019 | SMS campaign to 9,886 vehicles PE-CT. | M |
| 224 | 6/20/2019 | SMS campaign to 13,572 vehicles PE-CT. | M |
| 225 | 6/25/2019 | SMS campaign to 16,339 vehicles PE-CT. | M |
| 225 | 6/28/2019 | SMS campaign to 20,000 vehicles PE-CT. | M |
| 226 | 7/3/2019 | SMS campaign to 34,717 vehicles PE-CT. | M |
| 226 | 7/4/2019 | SMS campaign to 34,227 vehicles PE-CT. | M |
| 227 | 7/10/2019 | SMS campaign to 39,999 vehicles PE-CT. | M |
| 227 | 7/11/2019 | SMS campaign to 2,634 vehicles PPO-PDD. | M |
| 228 | 7/17/2019 | Mobile APP update for facilitating conditions (artificial intelligence-based assistant). | C |
| 228 | 7/19/2019 | Deadline for the declaration and payment. Mandate 43 of 17 December 2018, from the Departmental Assembly of Antioquia. | L |
| 229 | 7/24/2019 | SMS campaign to 62,306 vehicles PE-PDD. | M |
| 229 | 7/26/2019 | SMS campaign to 84,884 vehicles related to current term thanking payment. | M |
| 234 | 8/31/2019 | Web portal update for usability. | C |
| 236 | 9/9/2019 | Web portal update for reliability. | C |
| 301 | 1/7/2020 | SMS campaign to 250,964 vehicles PE-CT. | M |
| 302 | 1/9/2020 | SMS campaign to 80,000 vehicles PE-PDD. | M |
| 303 | 1/20/2020 | SMS campaign to 73,548 vehicles PE-CT. | M |
| 304 | 1/28/2020 | SMS campaign to 59,626 vehicles PE-CT. | M |
| 304 | 1/30/2020 | SMS campaign to 50,000 vehicles PE-CT. | M |
| 304 | 1/31/2020 | SMS campaign to 163,443 vehicles PE-CT. | M |
| 305 | 2/3/2020 | SMS campaign to 260,939 vehicles PE-CT. | M |
| 305 | 2/7/2020 | SMS campaign to 155,794 vehicles PE-CT. | M |
| 305 | 2/8/2020 | SMS campaign to 86,114 vehicles PE-PDD. | M |
| 306 | 2/10/2020 | SMS campaign to 65,036 vehicles PE-PDD. | M |
| 308 | 2/25/2020 | SMS campaign to 54,977 vehicles PE-CT. | M |
| 308 | 2/26/2020 | SMS campaign to 140,270 vehicles PE-CT. | M |
| 308 | 2/27/2020 | SMS campaign to 260,939 vehicles PE-CT. | M |
| 310 | 3/9/2020 | Advertising in social media (Google, Facebook, and Instagram) 14 days, promoting the payment. Ad clicks: 54,259. Visits to the web platform: 223,738. | M |
| 311 | 3/19/2020 | Departmental Decree 20200700001025 of 19 March 2020. All physical offices closed in preventative lockdown for COVID-19 pandemic. Only the web portal and mobile APP were available. | L |
| 311 | 3/20/2019 | Early payment deadline for 10% discount. Mandate 48 of 30 December 2019, from the Departmental Assembly of Antioquia. | L |
| 312 | 3/28/2020 | National Decree 491 of 28 March 2020. Preventive national lockdown for COVID-19 pandemic. The strict lockdown lasted until July 2020. | L |
| 320 | 5/20/2020 | National Decree 678 of 20 May 2020, establishing a 100% discount on interest and penalties, a 20% discount on capital for past due debts until 31 October 2020, and a 10% discount on capital for past due debts until 31 December 2020. | L |
| 326 | 6/29/2020 | Web portal update for usability. | C |
| 327 | 7/7/2020 | Social media (Google, Facebook, and Instagram) advertising for 11 days, promoting the payment. Ad clicks: 104,969. Visits to the web platform: 238,122. | M |
| 328 | 7/12/2020 | Medellín Municipal Decree 0706 of 12 July 2020, restricting access to the area La Candelaria (location of the main office in La Alpujarra) as a COVID-19 protective measure. | L |
| 328 | 7/17/2020 | Deadline for the declaration and payment. Mandate 48 of 30 December 2019, from the Departmental Assembly of Antioquia | L |
| 330 | 7/29/2020 | Mobile APP update for usability. | C |
| 340 | 10/6/2020 | Social media (Google, Facebook, and Instagram) advertising for 13 days, promoting the discounts. Ad clicks: 14,299. Visits to the web platform: 112,054. | M |
| 343 | 10/31/2020 | Deadline for 100% past due discount on interest and penalties and 20% discount on capital (National Decree 678 of 20 May 2020). | L |
| 347 | 11/25/2020 | Social media (Google, Facebook, and Instagram) advertising for 37 days, promoting the discounts. Ad clicks: 35,735. Visits to the web platform: 133,714. | M |
| 352 | 12/31/2020 | Deadline for 100% past due payment discount on interest and penalties, and 10% discount on capital (National Decree 678 of 20 May 2020). | L |
