# Supplementary material for: Government influence on e-government adoption by citizens in Colombia: Empirical evidence in a Latin American context
Source: PLoS One. 2022 Feb 25;17(2):e0264495. doi: 10.1371/journal.pone.0264495 (PMC8880567; doi:10.1371/journal.pone.0264495)
Supplement: S1 Scripts — (DOCX) [file pone.0264495.s003.docx]

import pandas as pd

import numpy as np

import statsmodels.api as sm

import statsmodels.formula.api as smf

data_antioquia=pd.read_excel('Data Weekly analysis(colab).xlsx', sheet_name='Payment and Filing 2015-2020')

#DEFINING THE MODEL

Xs=["MOBILIZATION","SW_UPD_ACUM","PAYMENT","LAWS_REGUL","COVID19"]

X=data_antioquia[Xs]

X=sm.add_constant(X)

#Y = E-PAYMENT ADOPTION

y=data_antioquia.PAY_EGOV_ADOPTION

#OLS REGRESSION

model=sm.OLS(y,X)

results = model.fit()

print(results.summary())

OLS Regression Results

==============================================================================

Dep. Variable: PAY_EGOV_ADOPTION R-squared: 0.722

Model: OLS Adj. R-squared: 0.717

Method: Least Squares F-statistic: 159.2

Date: Thu, 16 Dec 2021 Prob (F-statistic): 5.01e-83

Time: 12:03:09 Log-Likelihood: -1109.8

No. Observations: 313 AIC: 2232.

Df Residuals: 307 BIC: 2254.

Df Model: 5

Covariance Type: nonrobust

================================================================================

coef std err t P>|t| [0.025 0.975]

--------------------------------------------------------------------------------

const 21.8467 2.421 9.024 0.000 17.083 26.611

MOBILIZATION -1.2302 2.595 -0.474 0.636 -6.337 3.877

SW_UPD_ACUM 1.5594 0.127 12.307 0.000 1.310 1.809

PAYMENT -10.4630 2.060 -5.078 0.000 -14.517 -6.409

LAWS_REGUL 6.7654 2.523 2.682 0.008 1.802 11.729

COVID19 26.2523 3.173 8.274 0.000 20.009 32.495

==============================================================================

Omnibus: 40.801 Durbin-Watson: 0.639

Prob(Omnibus): 0.000 Jarque-Bera (JB): 226.572

Skew: 0.298 Prob(JB): 6.32e-50

Kurtosis: 7.125 Cond. No. 44.9

==============================================================================

Warnings:

[1] Standard Errors assume that the covariance matrix of the errors is correctly specified.

#ROBUST REGRESSION

model=sm.OLS(y,X)

results = model.fit()

#Heteroskedasticity and Autocorrelation Consistent (HAC)

new = results.get_robustcov_results(cov_type='HAC',maxlags=1)

print(new.summary())

OLS Regression Results

==============================================================================

Dep. Variable: PAY_EGOV_ADOPTION R-squared: 0.722

Model: OLS Adj. R-squared: 0.717

Method: Least Squares F-statistic: 64.07

Date: Thu, 16 Dec 2021 Prob (F-statistic): 1.22e-45

Time: 12:06:18 Log-Likelihood: -1109.8

No. Observations: 313 AIC: 2232.

Df Residuals: 307 BIC: 2254.

Df Model: 5

Covariance Type: HAC

================================================================================

coef std err t P>|t| [0.025 0.975]

--------------------------------------------------------------------------------

const 21.8467 2.311 9.452 0.000 17.299 26.395

MOBILIZATION -1.2302 3.508 -0.351 0.726 -8.133 5.673

SW_UPD_ACUM 1.5594 0.135 11.589 0.000 1.295 1.824

PAYMENT -10.4630 1.762 -5.939 0.000 -13.929 -6.996

LAWS_REGUL 6.7654 2.135 3.169 0.002 2.565 10.966

COVID19 26.2523 6.877 3.817 0.000 12.720 39.785

==============================================================================

Omnibus: 40.801 Durbin-Watson: 0.639

Prob(Omnibus): 0.000 Jarque-Bera (JB): 226.572

Skew: 0.298 Prob(JB): 6.32e-50

Kurtosis: 7.125 Cond. No. 44.9

==============================================================================

Warnings:

[1] Standard Errors are heteroscedasticity and autocorrelation robust (HAC) using 1 lags and without small sample correction

#GLS REGRESSION

model=sm.GLM(y, X, family=sm.families.Tweedie(var_power=1.8, eql=False))

results = model.fit()

print(results.summary())

Generalized Linear Model Regression Results

==============================================================================

Dep. Variable: PAY_EGOV_ADOPTION No. Observations: 313

Model: GLM Df Residuals: 307

Model Family: Tweedie Df Model: 5

Link Function: log Scale: 0.33690

Method: IRLS Log-Likelihood: nan

Date: Wed, 22 Dec 2021 Deviance: 250.85

Time: 16:36:46 Pearson chi2: 103.

No. Iterations: 12

Covariance Type: nonrobust

================================================================================

coef std err z P>|z| [0.025 0.975]

--------------------------------------------------------------------------------

const 3.1506 0.129 24.437 0.000 2.898 3.403

MOBILIZATION -0.0093 0.126 -0.074 0.941 -0.256 0.237

SW_UPD_ACUM 0.0923 0.006 14.369 0.000 0.080 0.105

PAYMENT -0.7490 0.110 -6.786 0.000 -0.965 -0.533

LAWS_REGUL 0.4064 0.126 3.222 0.001 0.159 0.654

COVID19 0.2815 0.153 1.837 0.066 -0.019 0.582

================================================================================

#Y = E-FILING

y=data_antioquia.EFILING_EGOV_ADOPTION

#OLS REGRESSION

model=sm.OLS(y,X)

results = model.fit()

print(results.summary())

OLS Regression Results

=================================================================================

Dep. Variable: EFILING_EGOV_ADOPTION R-squared: 0.345

Model: OLS Adj. R-squared: 0.335

Method: Least Squares F-statistic: 32.40

Date: Thu, 16 Dec 2021 Prob (F-statistic): 1.68e-26

Time: 12:14:56 Log-Likelihood: -1272.5

No. Observations: 313 AIC: 2557.

Df Residuals: 307 BIC: 2579.

Df Model: 5

Covariance Type: nonrobust

================================================================================

coef std err t P>|t| [0.025 0.975]

--------------------------------------------------------------------------------

const 49.3419 4.072 12.118 0.000 41.330 57.354

MOBILIZATION 2.6855 4.365 0.615 0.539 -5.903 11.274

SW_UPD_ACUM 1.9364 0.213 9.087 0.000 1.517 2.356

PAYMENT 1.8944 3.465 0.547 0.585 -4.924 8.713

LAWS_REGUL 14.9517 4.242 3.524 0.000 6.604 23.300

COVID19 -3.7885 5.336 -0.710 0.478 -14.288 6.711

==============================================================================

Omnibus: 146.591 Durbin-Watson: 0.517

Prob(Omnibus): 0.000 Jarque-Bera (JB): 594.441

Skew: -2.046 Prob(JB): 8.30e-130

Kurtosis: 8.369 Cond. No. 44.9

==============================================================================

Warnings:

[1] Standard Errors assume that the covariance matrix of the errors is correctly specified.

#ROBUST REGRESSION

model=sm.OLS(y,X)

results = model.fit()

#Heteroskedasticity and Autocorrelation Consistent (HAC)

new = results.get_robustcov_results(cov_type='HAC',maxlags=1)

print(new.summary())

OLS Regression Results

=================================================================================

Dep. Variable: EFILING_EGOV_ADOPTION R-squared: 0.345

Model: OLS Adj. R-squared: 0.335

Method: Least Squares F-statistic: 51.44

Date: Thu, 16 Dec 2021 Prob (F-statistic): 1.21e-38

Time: 12:16:17 Log-Likelihood: -1272.5

No. Observations: 313 AIC: 2557.

Df Residuals: 307 BIC: 2579.

Df Model: 5

Covariance Type: HAC

================================================================================

coef std err t P>|t| [0.025 0.975]

--------------------------------------------------------------------------------

const 49.3419 6.218 7.935 0.000 37.106 61.578

MOBILIZATION 2.6855 1.903 1.411 0.159 -1.059 6.430

SW_UPD_ACUM 1.9364 0.231 8.378 0.000 1.482 2.391

PAYMENT 1.8944 4.514 0.420 0.675 -6.988 10.777

LAWS_REGUL 14.9517 2.824 5.295 0.000 9.395 20.508

COVID19 -3.7885 3.259 -1.162 0.246 -10.202 2.624

==============================================================================

Omnibus: 146.591 Durbin-Watson: 0.517

Prob(Omnibus): 0.000 Jarque-Bera (JB): 594.441

Skew: -2.046 Prob(JB): 8.30e-130

Kurtosis: 8.369 Cond. No. 44.9

==============================================================================

Warnings:

[1] Standard Errors are heteroscedasticity and autocorrelation robust (HAC) using 1 lags and without small sample correction

#GLS REGRESSION

model=sm.GLM(y, X, family=sm.families.Tweedie(var_power=1.8, eql=False))

results = model.fit()

print(results.summary())

Generalized Linear Model Regression Results

=================================================================================

Dep. Variable: EFILING_EGOV_ADOPTION No. Observations: 313

Model: GLM Df Residuals: 307

Model Family: Tweedie Df Model: 5

Link Function: log Scale: 0.15754

Method: IRLS Log-Likelihood: nan

Date: Wed, 22 Dec 2021 Deviance: 288.36

Time: 16:53:37 Pearson chi2: 48.4

No. Iterations: 12

Covariance Type: nonrobust

================================================================================

coef std err z P>|z| [0.025 0.975]

--------------------------------------------------------------------------------

const 3.8750 0.076 51.192 0.000 3.727 4.023

MOBILIZATION 0.0407 0.079 0.515 0.607 -0.114 0.196

SW_UPD_ACUM 0.0327 0.004 8.366 0.000 0.025 0.040

PAYMENT 0.0582 0.064 0.905 0.365 -0.068 0.184

LAWS_REGUL 0.2445 0.077 3.174 0.002 0.094 0.396

COVID19 -0.0967 0.096 -1.003 0.316 -0.286 0.092

================================================================================
