# Supplementary material for: Government influence on e-government adoption by citizens in Colombia: Empirical evidence in a Latin American context
Source: PLoS One. 2022 Feb 25;17(2):e0264495. doi: 10.1371/journal.pone.0264495 (PMC8880567; doi:10.1371/journal.pone.0264495)
Supplement: Attachment — Submitted filename: Comments_and_responses.docx [file pone.0264495.s004.docx]

PONE-D-21-27851
Government influence on e-government adoption by citizens in Colombia: An exploratory analysis in a Latin American context
PLOS ONE

Dear Dr. Juan Pablo,

Thank you for submitting your manuscript to PLOS ONE. After careful consideration, we have decided that your manuscript does not meet our criteria for publication and must therefore be rejected.

I am sorry that we cannot be more positive on this occasion, but hope that you appreciate the reasons for this decision.

Yours sincerely,

Rogis Baker, Ph.D
Academic Editor
PLOS ONE

***Response:*** *Thank you very much for your valuable comments. We have updated the document in accordance with the recommendations and comments. We have published the data in openICPSR. Moreover, we are sharing with you the SPSS file, the log of the analysis executed, and an Excel file with the analyzed information and the catalog.*

**Reviewers' comments**

Reviewer's Responses to Questions

**Comments to the Author**

1. Is the manuscript technically sound, and do the data support the conclusions?

The manuscript must describe a technically sound piece of scientific research with data that supports the conclusions. Experiments must have been conducted rigorously, with appropriate controls, replication, and sample sizes. The conclusions must be drawn appropriately based on the data presented.

Reviewer #1: Yes

Reviewer #2: Yes

Reviewer #3: Partly

2. Has the statistical analysis been performed appropriately and rigorously?

Reviewer #1: Yes

Reviewer #2: I Don't Know

Reviewer #3: No

3. Have the authors made all data underlying the findings in their manuscript fully available?

The [PLOS Data policy](http://www.plosone.org/static/policies.action#sharing) requires authors to make all data underlying the findings described in their manuscript fully available without restriction, with rare exception (please refer to the Data Availability Statement in the manuscript PDF file). The data should be provided as part of the manuscript or its supporting information, or deposited to a public repository. For example, in addition to summary statistics, the data points behind means, medians and variance measures should be available. If there are restrictions on publicly sharing data—e.g. participant privacy or use of data from a third party—those must be specified.

Reviewer #1: No

Reviewer #2: No

Reviewer #3: Yes

4. Is the manuscript presented in an intelligible fashion and written in standard English?

PLOS ONE does not copyedit accepted manuscripts, so the language in submitted articles must be clear, correct, and unambiguous. Any typographical or grammatical errors should be corrected at revision, so please note any specific errors here.

Reviewer #1: Yes

Reviewer #2: Yes

Reviewer #3: Yes

5. Review Comments to the Author

Please use the space provided to explain your answers to the questions above. You may also include additional comments for the author, including concerns about dual publication, research ethics, or publication ethics. (Please upload your review as an attachment if it exceeds 20,000 characters)

**Reviewer #1:**

The research paper aims to identify government influence over the adoption of e-government by citizens, and identifies coercive pressure, conformance to the environment, and knowledge deployment as important determinants of adoption of e-government by citizens.

The research intent of the paper is original insofar as the geographical focus of the case study, i.e. Antioquia Colombia.

A comprehensive review of institutional theory literature focusing on e-government adoption provides theoretical framework for understanding research from authors' specific perspective. However, the authors fail to include more fundamental institutional theory scholars' work (e.g. Moe 1984; March and Olsen, 1989) which reduces the quality and scope of the literature review.

***Response****: Thank you very much for your valuable comments. In this new version we have complemented the theoretical framework with the referenced works (page 6, paragraph 4).*

Despite the acknowledged lack of generalizability, this research does serve as a foundation or plan upon which future research can build by looking at additional variables, within various populations as the methods chosen can be easily reproduced in various contexts.

Quality of images need to be enhanced. In addition, the data and code that has been run, as well as the output, should be made available so that they can be considered when the methodology and results section are being reviewed.

***Response:*** *We have enhanced the quality of the images. In addition, we have published the data in openICPSR. Also, we are sharing with you the SPSS file, the log of the analysis executed, and an Excel file with the analyzed information and the catalog.*

Overall, the paper is very well-written and structured, and the mixed-methods approach taken is appropriate, as the interviews and data from various laws, regulations and digital and physical records provided needed context to the statisitical findings of the study.

**Reviewer #2 - Recommendation:**

Thanks for the opportunity to review the manuscript "Government influence on e-government adoption by citizens in Colombia: An exploratory analysis in a Latin American context" (PONE-D-21-27851).

The logic of the paper is very unclear and we cannot understand the point the authors are trying to make. I could not understand at all what the case study and the empirical study were trying to say and how they were connected to the previous analysis.

Also, the format of the paper makes it very difficult for me to read.

***Response:*** *Thank you for your time and comments. According to your recommendation, we submitted the document to an editing services and have included the certificate in this submission. Complementarily, let us give you a summary of this study:*

*This research paper aims to identify government influence over the adoption of e-government by citizens (AEC), and identifies coercive pressure, conformance to the environment, and knowledge deployment as important determinants of AEC.

- For the dependent variable (AEC), we analyzed the weekly consolidated data from 2015 to 2020 (more than 11 million filing records and more than 5 million payments, represented over 313 weeks), including service type (e-filing, e-payment), week, number of services for overdue and current term per channel (current term physical, current term e-government, due term physical, and due term e-government). We studied the behavior of the weekly AEC rate.*

*- In parallel, to provide context for the data, we collected data from physical records and documents, digital records, and relevant laws and regulations. We conducted several interviews with the service operation manager and staff from 2018 to 2021 to identify all actions related to the services performed for three years, starting from 1 January 2018 to 31 December 2020. Each action performed during the three years was classified according to the institutional factor groups, used as independent variables, and analyzed. We related these actions with the AEC rate behavior. For context, the findings were discussed with the staff. All the data from different sources support the credibility of the findings since they allow triangulation and capture contextual complexity.*

*- We employed institutional theory, institutional interventions, and legitimation strategies to classify these actions, used as independent variables. In summary, we observed actions for knowledge deployment, conformance to the environment (fulfilling demand-side expectations with software updates, quality of service, and quality of software), mobilization, coercive pressure (laws and regulations for due dates, discounts and penalties, and measures in 2020 related to COVID-19).*

*- We analyzed the correlation of these actions with AEC for two services from 2015 to 2020 using multiple linear regression for data correlation.*

**Reviewer #3: Review for PLOS ONE**
Manuscript: Government influence on e-government adoption by citizens in Colombia: An exploratory analysis in a Latin American context

PONE-D-21-27851

This study examines focuses on e-government and whether the government itself can in influence their citizens to use such facilities in Antioquia, Colombia. This study is particularly timely considering the need of individuals for services that do not require a face-to-face interaction. Below I provide some suggestions that could improve the quality of the paper.

The authors could highlight in their motivation the need for online services and what are the benefits in doing so. To this end, it is also necessary to acknowledge that not all citizens would benefit from such services as some may not have access to the internet (at home) or they do not know how to use such services (e.g., the elderly). A more thorough discussion on demand is necessary, but this should be linked to the supply of the internet and ICT in general.

***Response:*** *Thank you very much for your valuable comments. This new version of the document includes these subjects in the introduction (page 4), the case study (pages 16–17), and the discussion (page 25).*

I appreciate the authors recognise that we can’t assume the use of AEC by default just because they exist but there are some additional contribution factors that are not taken into consideration here beyond the availability. Take for instance, how user friendly some of these e-government services are. Although there is a related question in the analysis, I did not see this point discussed in the theoretical framework. Citizens may still opt for an in person interaction because they are either used to this or they do not want to spend the time to familiarise themselves with the online environment. This means that the government’s efforts in promoting the service are likely to be dependent on the quality of the AEC.

***Response****: We have included this discussion in the literature review (page 6). Also, on page 21, we reference how important the quality of service has been for AEC in our case study. Finally, in the discussion (page 26), the relevance of the quality of technical solutions and service is highlighted.*

This discussion should be expanded on the selection of the case study. The authors have chosen a country that was “the Latin American leader in the UN e-government survey (37) in 2010 due to 122 the unique strategies of its government. I wonder whether it is possible to make any generalisations of the findings for other Latin American countries or other countries outside Latin America. The authors could elaborate on the limited scope conditions of the study. Again, the focus of the authors is on the supply of the services but I consider important to discuss the demand and accessibility as well.

***Response****: We have included references to the Latin American context in the case study (page 16–17), discussion (page 25), and limitations (pages 28–29).*

I find the discussion on legitimacy too general and not very much linked to the e-government services. Additionally, the point on legitimacy between democracies and autocracies is rather distracting from the focus here.

***Response****: We have modified this section according to your recommendations (page 8).*

It would be informative to know whether the e-government services analysed are a product of the government analysed.

***Response****: In the case study section (page 17), we have mentioned that these services are products of the government analyzed, and that the government has total control of them.*

I recommend the authors to consider selection effects in their empirical analysis, particularly regarding the covid-19 indicator. During this period many in-person services may have been ceased and thus online interactions were the only available ones.

***Response****: We have reviewed our selection effects in the empirical analysis. We have included references to the limitations generated by the Covid-19 lockdown measures and the unavailability of physical offices in the discussion (page 25). However, we found that the effects reflect the coercive pressure exerted by the government, limiting the access to the traditional option and directly and favoring the use of the digital channel. This is the same effect that the Danish government obtained from the mandatory use of digital channels.*

The authors discuss the Danish case as a comparison to their findings and this goes back to my earlier comment on generalization of their findings.

***Response****: Thank you for your valuable input. You were right. We have included references to the Latin American context in the case study (page 16–17), discussion (page 25), and limitations (page 28–29).*
